# Composite Scaffolds Containing Silk Fibroin, Gelatin, and Hydroxyapatite for Bone Tissue Regeneration and 3D Cell Culturing

**Published:** 2014

**Authors:** M. M. Moisenovich, A. Yu. Arkhipova, A. A. Orlova, M. S Drutskaya, S. V. Volkova, S. E. Zacharov, I. I. Agapov, M. P. Kirpichnikov

**Affiliations:** Biological Faculty, Moscow State University, Leninskie Gory, 1-12, 119991, Moscow, Russia; Shumakov Institute of Transplantology and Artificial Organs, Federal Agency for High-Tech Medical Services, Shchukinskaya Str., 1, 113182, Moscow, Russia

**Keywords:** adhesion, hydroxyapatite, gelatin, composite biodegradable scaffolds, proliferation, silk fibroin

## Abstract

Three-dimensional (3D) silk fibroin scaffolds were modified with one of the major bone tissue derivatives (nano-hydroxyapatite) and/or a collagen derivative (gelatin). Adhesion and proliferation of mouse embryonic fibroblasts (MEF) within the scaffold were increased after modification with either nano-hydroxyapatite or gelatin. However, a significant increase in MEF adhesion and proliferation was observed when both additives were introduced into the scaffold. Such modified composite scaffolds provide a new and better platform to study wound healing, bone and other tissue regeneration, as well as artificial organ bioengineering. This system can further be applied to establish experimental models to study cell-substrate interactions, cell migration and other complex processes, which may be difficult to address using the conventional two-dimensional culture systems.

## INTRODUCTION

Developing and improving the techniques for the restoration of damaged or lost organs and tissue fragments, as well as constructing artificial organs, are pressing issues in tissue engineering and regenerative medicine today. Low-immunogenicity biomaterials that can maintain cell adhesion and proliferation, and degrade to their chemical derivatives safe for the organism with time, are required for a technological breakthrough in these fields. Bacterial polyhydroxyalkanoates are an example of such advanced materials [1]. An important advantage of these materials is that they exhibit unique mechanical properties, plasticity, and tolerance to extrusion processing. Bacterial polyhydroxyalkanoates can be used to manufacture irregularly shaped items; hence, they are a rather promising material for 3D prototypes. These materials are characterized by a lower biocompatibility compared to collagen and other extracellular matrix components. However, the use of collagen is limited by its mechanical properties, while articles made of silk fibroin demonstrate a good biocompatibility, along with high mechanical resistance and elasticity. The availability of silk, its water solubility, biodegradability with the formation of amino acids, thermal resistance, the availability of easily accessible chemical groups for functional modification, radioresistance, the possibility of using gas sterilization, and suitability for composite materials are additional important benefits [2, 3]. The increasing number of publications and references on the use of fibroin for the re-generation of various organs and tissues (tendons, ligaments, cartilages, bone tissue, skin, liver, trachea, nerves, retina, tympanic membrane, and bladder) attests to the high potential of the polymer as a material for biomedicine [4].

We compared the properties of scaffolds from fibroin and recombinant spidroin in our previous studies. Those studies showed that re-generated fibroin maintains the adhesion and proliferation of fibroblasts (one of the main components involved in wound healing and tissue regeneration) to a lesser extent than the substrate formed by polymerized recombinant spidroin from *Nephila clavipes*. The reduced capability of fibroin materials to maintain cell adhesion and proliferation has the potential to cause a poorer re-generation ability compared with that of spidroin scaffolds in experiments with a bone injury model. The re-generative properties of fibroin scaffolds in these experiments were considerably improved by the use of nano-hydroxyapatite mineralization [5]. We have introduced a combination of two composite additives, nano-hydroxyapatite (a bone tissue component) and gelatin (a collagen derivative), into the formulations of fibroin scaffolds to enhance their capability to maintain the adhesion and proliferation of fibroblasts. The composite substrate formed by all three components was the optimal material that maintained MEF adhesion and proliferation.

## EXPERIMENTAL

Pods of bombycid, *Bombyx mori*, were kindly provided by V.V. Bogoslovskii, Director of the Republican Sericulture Research Station of the Russian Academy of Agricultural Sciences (Zheleznovodsk, Stavropol region). The desericinization technique was used to produce pure fibroin. Sericin and other impurities were removed from the pods by boiling in a 0.03 M NaHCO_3_ solution (pH 8.4) for 1.5 h, followed by washing with water and drying. Natural hydroxyapatite was provided by Prof. V.V. Guzeev (Seversk Technological Institute, National Research Nuclear University MEPhI, Russia).

**Scaffold Manufacturing**

To manufacture a scaffold, a weighted fibroin sample (250 mg) was dissolved in 1,000 µL of a 10% lithium chloride solution in 90% formic acid at 60–70oC for 30 min. A mixture containing fibroin (225 mg) and gelatin (25 mg) in 1,000 µL of the solution was used to form a composite scaffold with a 10% content of gelatin. The resulting solution was centrifuged at 12,100 *g* for 5 min; the supernatant was used to form scaffolds. 50 µL of the pre-heated supernatant was placed into the mold, layer-by-layer, and mixed with 100 mg of sodium chloride with different particle sizes. NaCl crystals (150–300 µm in diameter) were used as an expanding agent. A weighted sample of HA powder was mixed with expanding NaCl particles (150–300 µm in diameter) to produce composite scaffolds with a 30% HA content. The salt concentration was selected in such a manner as to form a scaffold with a complex internal porous surface free of isolated cavities. The resulting samples were dried at 75–80°C for 3 h, kept at ambient temperature for 16 h, processed with 96% ethanol for 120 min, washed in bidistilled water for 120 min, and degased and stored in 70% ethanol.

**Scanning Electron Microscopy (SEM)**

Scanning electron microscopy was used to examine the structure of the scaffolds.

SEM samples were prepared by the standard procedures: fixation in glutaric aldehyde and dehydration in graded series of ethanol and acetone. The samples were then dried by the critical point method in an HCP-2 critical point dryer (Hitachi Ltd., Japan). The samples were sputter-coated with a 20 nm-thick layer of gold in an argon atmosphere with a 6 mA ion current and 0.1 mm Hg in an Ion Coater IB-3 (Eiko Engineering, Mito, Japan). A Camscan S2 microscope (Cambridge Instruments, Cambridge, UK) with a 10 nm resolution and 20 kV operating volume was used (the SEI mode) for scanning electron microscopy. The MicroCapture software (SMA, Russia) was used to capture images.

**Confocal Laser Scanning Microscopy (CLSM)**

We used a confocal laser scanning system (Nikon, Japan) in which Eclipse, a clinical inverted microscope for laboratory studies, is combined with an A1 confocal module. The pinhole size, laser parameters, and analyzing filter size for all series of optical sections were chosen as recommended by the manufacturer to achieve a high resolution of the images.

**Primary Cultures of the GFP Expressing Mouse Embryonic Fibroblasts**

MEF cells were isolated from GFP+ embryos on the 13.5th day of intrauterine growth. Two C57Bl/6 females were mated with a GFP+ male for a night and checked for vaginal plugs the next morning. The moment of plug detection was considered to be the 0.5^th^ day of time-dated pregnancy. The mice were euthanized on the 13.5^th^ day of pregnancy. The uterus was removed; heads and internals were separated from the embryos, and GFP expression was determined using a trans-illuminator. The rest of the tissues were aseptically chopped with eye scissors, dissociated in a 0.05% trypsin/EDTA solution, and centrifuged at 1,000 rpm for 5 min. The resulting cell suspension was transferred into 25-cm^2^ cultural flasks for adherent cell growth (Greiner). The cells were subsequently cultivated in DMEM supplemented with 4.5 g/L glucose (HyClone) and 10% fetal bovine serum (HyClone) at 37°C, 5% CO_2_, and 95% humidity. The cells were passaged at a 1:3 ratio every three days after they reached 80-85% confluence. The C57Bl/6 females were purchased from the Pushchino Animal Breeding Facility (BIBC RAS); and the transgene males with the expressed GFP were kindly provided by N.N. Logunova (ISTC RAMS).

The C57Bl/6 females were purchased from the Pushchino Animal Breeding Facility (BIBC RAS); and the transgene males with the expressed GFP were kindly provided by N.N. Logunova (ISTC RAMS).

## RESULTS AND DISCUSSION

**Fig. 1:**
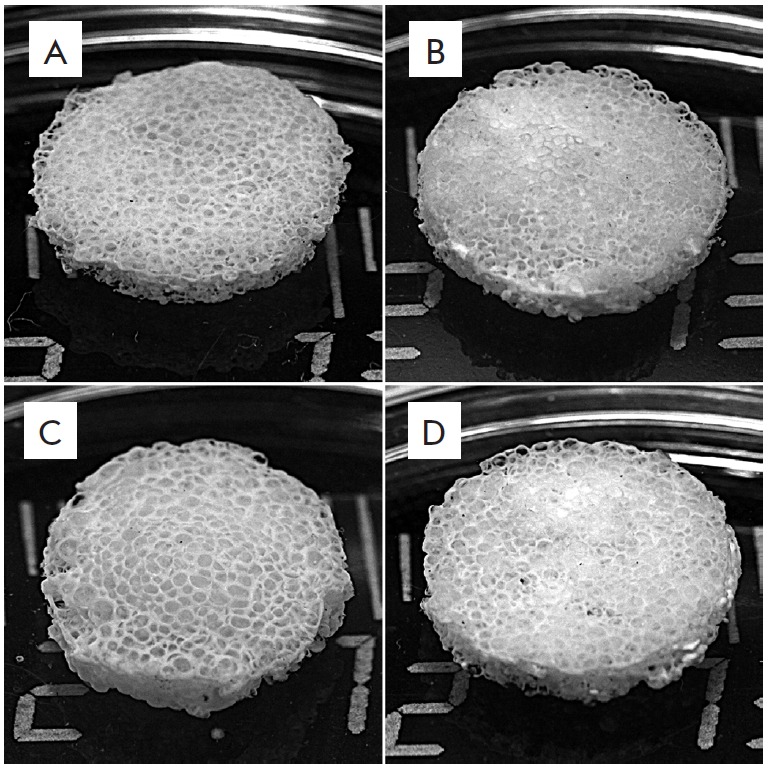
Appearance of 3D porous silk fibroin (A) and composite fibroin–gelatin (B), fibroin–hydroxyapatite (C), and fibroin–gelatin–hydroxyapatite (D) scaffolds. Introduction of gelatin and hydroxyapatite into the scaffold structure does not modify its appearance

We had previously formed silk fibroin scaffolds [6] and silk fibroin–HA scaffolds [5], and examined the biological properties of the pilot samples. The scaffolds possess all the characteristics needed for bone surgery; in particular, they are biocompatible, strong, and porous. The current study yielded silk fibroin scaffolds, composite silk fibroin–gelatin and silk fibroin–HA scaffolds, and composite scaffolds containing three main components: silk fibroin, gelatin, and HA (*Fig. 1*). A pore-forming agent with a preset particle diameter was selected to produce these scaffolds.

The resulting test samples could maintain their integrity and acquired the preset cylindrical shape. The composite silk fibroin–gelatin scaffolds underwent an elastic deformation under direct mechanical pressure, while the silk fibroin–HA scaffolds remained un-deformed. The pores of the scaffolds produced by leaching had sizes corresponding to the added particles of the pore-forming agent (150–300 µm).

**Fig. 2:**
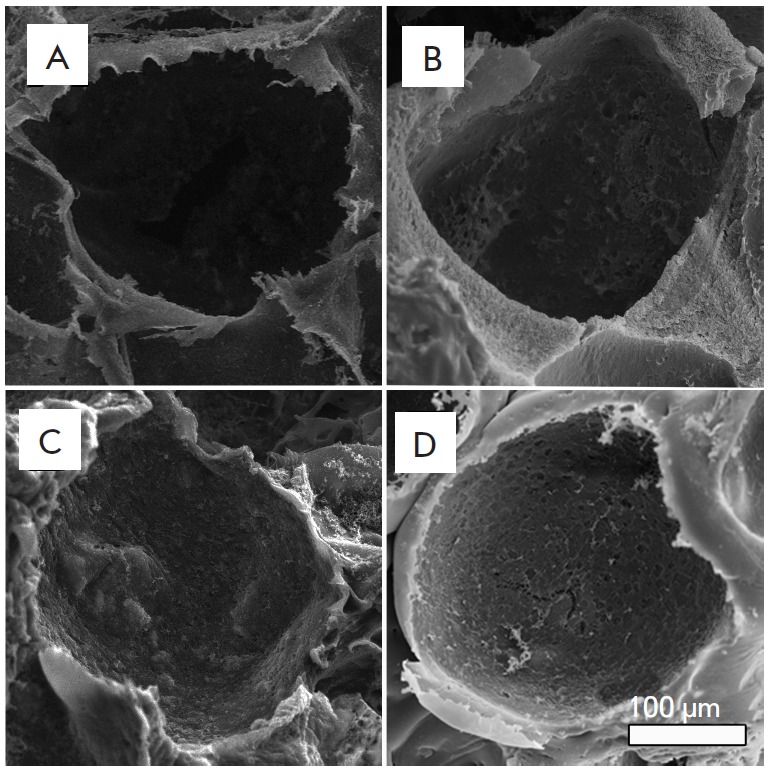
Structure of 3D porous silk fibroin (A) and composite fibroin–gelatin (B), fibroin–hydroxyapatite (C), and fibroin–gelatin–hydroxyapatite (D) scaffolds. The images were recorded on a scanning electron microscope. Introduction of gelatin and hydroxyapatite into the scaffold structure does not modify the pore size and the general scaffold structure

**Fig. 3:**
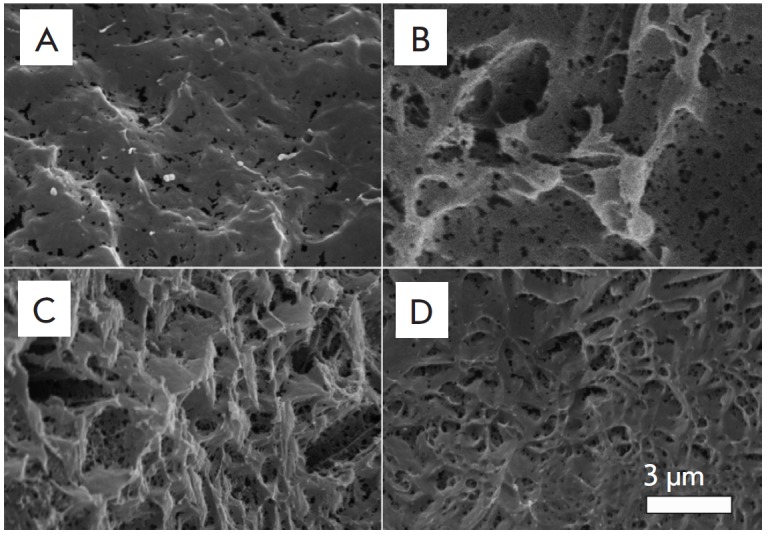
Pore wall surface of silk fibroin (A) and composite fibroin–gelatin (B), fibroin–hydroxyapatite (C), and fibroin–gelatin–hydroxyapatite (D) scaffolds. The images were recorded on a scanning electron microscope. Introduction of gelatin and hydroxyapatite into the scaffold structure changes the fine architecture of the scaffolds

The surface of the products was examined by scanning electron microscopy (SEM) (*Fig. 2*). The resulting scaffolds had a cellular mesh structure totally free of the pore-forming agent (its traces were never found in the material) (*Figs. 2, 3*). The permeability test with suspended colored ink particles confirmed the conjunctivity of the scaffold pores.

The examination of the sample structure showed that the amounts of gelatin and HA in a composite scaffold did not affect the conjunctivity of the pores, appearance of the articles, and their ink permeability. Three test samples had the same porosity and appearance due to the fact that substance porosity is governed by the parameters of the pore-forming agent (which forms pores 150–300 µm in diameter) and is independent of the amount of additives, gelatin, or HA.

The pore diameter dictates the mechanical properties of a structure and the rate of its biodegradation; it also affects the post-implantation tissue response and cell interaction with the scaffold surface. Larger pores facilitate a better and more rapid integration of the newly formed tissue, its vascularization, and a more effective bioresorption of a graft.

Three-dimensional cell culturing requires scaffolds with an unclosed structure. Pores connected with holes and channels form a complex, unclosed internal surface that facilitates cell migration to the internal layers of an artificial scaffold. Furthermore, an unclosed pore structure provides conditions for the medium exchange and removal of metabolites, thus facilitating the formation of a homogenous intra-scaffold medium [5, 7-9].

CLSM examination showed that a water medium affects the integrity and porosity of both the fibroin and all composite scaffolds neither immediately after immersion (1 h) nor a day later. This characteristic is very important, since disintegration or alteration of the basic structure and physical characteristics of a graft in a water medium prevents its use *in vivo*. Lack of considerable water-absorbing and water-retaining abilities allowed the articles to keep their preset parameters.

Adhesion of substrate-dependent cells on the scaffold surface is neccesary to maintain their viability in a 3D culture [10, 11]. A substrate affects the production of extracellular matrix components by the cells, its synthesis, and composition. The ability to maintain cellular adhesion and proliferation is considered to be an important *in vitro *biocompatibility parameter for a material used as a substrate [10-12]. Hence, a material with inhibiting properties will inhibit tissue regeneration* in vivo*.

Silk fibroin is a high-strength protein free of carcinogenic, toxicogenic, or allergenic properties. It preserves its functional characteristics for a given period, causes no local inflammatory response, does not trigger the spread of an infection, and is replaced with a patient’s native tissue over time; therefore, it is a material suitable for bone tissue re-generation [5-7].

Fibroin is an amphiphilic protein with considerable prevalence of hydrophobic properties [13]; its isoelectric point p*I *is 4.2. Due to this parameter, it is soluble neither in water nor in the diluted solutions of some acids and bases [13], while it is negatively charged at physiological pH=7, in contrast to the positively charged spidroin [5], thus decreasing cell adhesion and increasing the cell proliferation rate [5].

A collagen derivative, gelatin, was used as an additive for composite materials. Collagen is the main fibrillar component of the extracellular matrix and connective tissue, with a molecular weight of 300 kDa. Collagen is found in almost all tissue types, ensuring their strength and structural stability. Thus, the protein comprises approximately 30% of the total protein mass in mammals. This material is not toxic and is a weak allergen; however, important shortcomings of collagen scaffolds include poor mechanical properties and short biodegradation time (it is regulated by cross-linking agents only partially, which limits the lifetime of collagen articles to one month). Gelatin is a product of collagen denaturation. It contains a large amount of glycine, proline, and 4-hydroxyproline, along with the three-amino-acid sequence (arginine, glycine, and aspartate – RGD), which bind to cell receptors (integrins), thus promoting cell adhesion and proliferation. Similar sequences are found in other proteins of the cell matrix; however, their use considerably increases the cost of these products.

We have examined the effects of scaffold additives on the adhesion and proliferation of primary MEF. Fi- Fig. 5. Increasing count of murine embryonic fibroblasts (MEF) during cultivation on 3D porous silk fibroin and composite scaffolds Number of cells in 1 mm2 view 1400 1200 1000 800 600 400 200 0 Time of cultivation, days 1 4 7 Fibroin Fibroin + Gelatin Fibroin + HA Fibroin + Gelatin + HA broblasts are a heterogeneous cell population capable of producing such extracellular matrix components as procollagen, fibronectin, proelastin, glucose aminoglycans, nidogen, laminin, tenastin, and chondroitin-2-sulfate. Fibroblasts take an active part in wound-healing and epithelization [14]. Moreover, they can secrete vascular epithelium growth factors (VEGF), thus stimulating angiogenesis and the formation of lymphatic vessels [15, 16]. We chose the primary culture of mouse embryonic fibroblasts, whose proliferative potential is higher than that in postnatal culture cells.

**Fig. 4:**
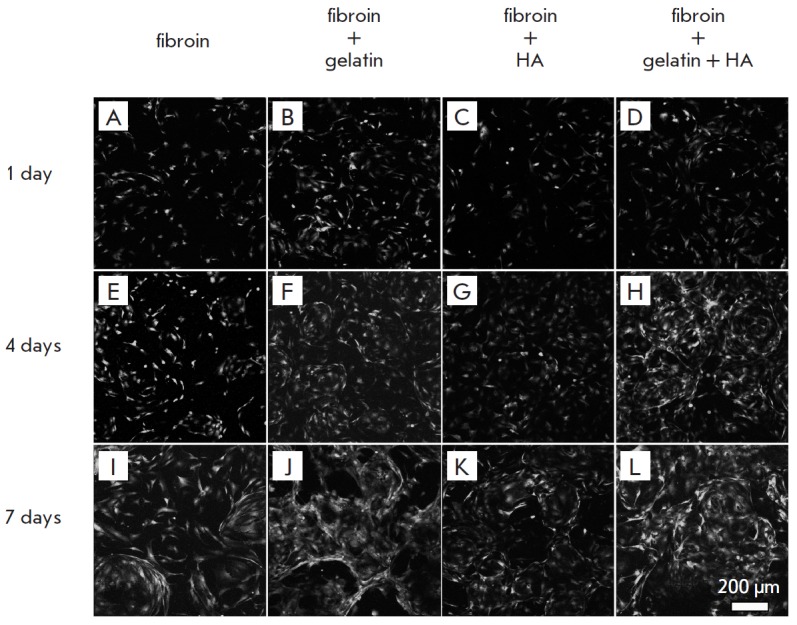
GFP-expressing murine embryonic fibroblasts (MEF) on the silk fibroin scaffold (A, E, I), composite fibroin–gelatin scaffold (B, F, J), hydroxyapatite (C, G, K), gelatin and hydroxyapatite (D, H, L) after 1 (A–D), 4 (E–H), and 7 (I–L) days of cultivation. The images show surface projections of the optical sections

**Fig. 5:**
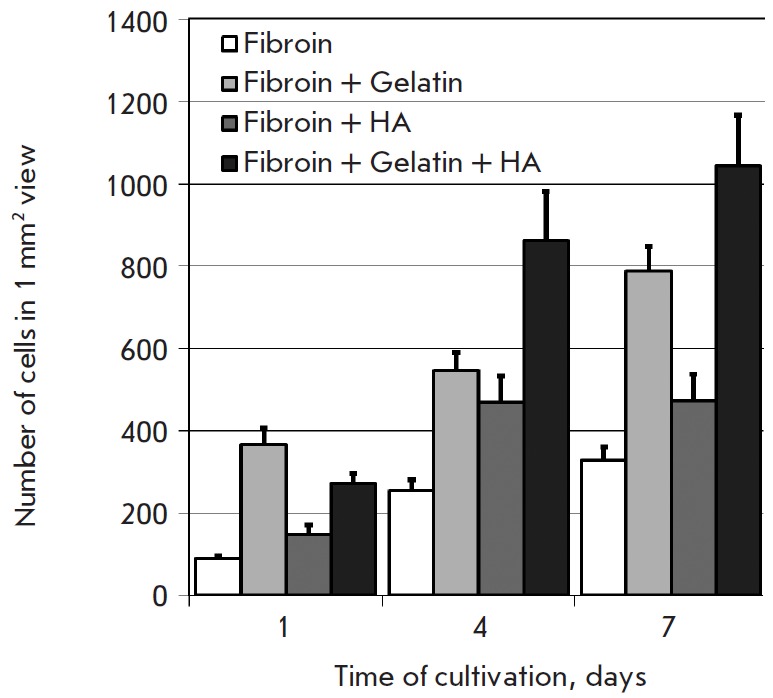
Increasing count of murine embryonic fibroblasts (MEF) during cultivation on 3D porous silk fibroin and composite scaffolds

The images recorded by CLSM are a series of horizontal optical sections of a scaffold. Cells and scaffold structures up to 300 µm deep were available (*Fig. 4*). The images were used for cell counting. The changes in the number of cells cultivated on different scaffolds over time were compared. The gelatin and HA introduced into the scaffold structure enhanced cell adhesion and the proliferative rate (*Fig. 5*). Thus, within a day, the cellcount on a composite scaffold was 2.5-fold greater than that on a fibroin scaffold, while on days 4 and 7, it increased more than threefold.

## CONCLUSIONS

Silk fibroin scaffolds and composite scaffolds with gelatin and HA additives were produced in this study. These scaffolds have an unclosed structure, maintain their integrity, and are not mechanically disintegrated. Modification of fibroin scaffolds with gelatin and HA simultaneously alters the properties of their surface. These alterations enhance MEF adhesion and proliferation in a 3D culture, making the modified scaffolds a promising material for regenerative medicine, especially for bone tissue regeneration.

## Abbreviations

GFP: green fluorescent protein
GRD: the one-letter amino acid abbreviation for Arginine-Glycine-Aspartic acid
HA: hydroxyapatite
CLSM: confocal laser scanning microscopy
MEF: murine embryonic fibroblasts
SEM: scanning electron microscopy
